# Respective healthcare system performances taking into account environmental quality: what are the re-rankings for OECD countries?

**DOI:** 10.1186/s12961-023-01005-6

**Published:** 2023-06-19

**Authors:** Armel Ngami, Bruno Ventelou

**Affiliations:** Aix Marseille Univ, CNRS, IRD, AMSE, Marseille, France, 5-9 Boulevard Bourdet, CS 50498, 13205 Marseille Cedex 1, France

**Keywords:** Health, Healthcare system efficiency, Health production function, Environment, Stochastic frontier analysis, Panel data

## Abstract

**Background:**

Efficiency analyses have been widely used in the literature to rank countries regarding their health system performances. However, little place has been given to the environmental aspect: two countries with the same characteristics could experience completely different healthcare system outcomes just because they do not face the same environmental quality situation, which is a major determinant of the health of inhabitants.

**Methods:**

Using a stochastic frontier model, this paper analyses the effect of environmental quality on health system outcomes in OECD countries, measured by life expectancy at birth.

**Results:**

We show that the healthcare system performance ranking of OECD countries changes significantly, depending on whether the environmental index is taken into account.

**Conclusions:**

These findings, once again, underline the critical importance of the environment when addressing population health issues. In general, our results can be aligned with the messages of the One Health approach literature.

## Introduction

Since the beginning of this century, there has been a growing body of literature on the issue of health system efficiency. This notion reflects the best way for a country to take advantage of several inputs or factors such as health infrastructures, number of nurses or physicians, the level of education and so on to produce the best health output for its population. With this in mind, countries could therefore be compared with respect to their “performance” (the extent to which the goal of an efficient use of inputs is achieved). For instance, in 2014, per capita, the United States of America was 35% richer and spent almost twice more on health than France, but the life expectancy at birth was almost 4 years longer in France than in the United States [1].

In the same period, the research devoted to the environment in health system performance studies is tiny, despite the extensive literature that documents the relationship between environment quality and population health status [2]. Pollution is one of the major causes of disease and was responsible for 9 million premature deaths around the world in 2015 [3]. Conversely, preserving ecosystems, such as green forests, extends life expectancy by improving air quality.

A study by WHO [4] in 2000 was the first to propose an assessment and ranking of all national health system performances [5]. The approach used was to estimate a fixed-effect panel data model, with education and health expenditure as health inputs, to assess time-invariant country health system inefficiency. This generated much debate and several authors afterwards suggested alternative methods, such as data envelopment analysis [6] or stochastic frontier analysis [7], to correct the shortcomings of the WHO seminal study.

Nevertheless, none of these studies includes environmental quality as a factor in the process of creating and maintaining population health. In addition, ranking the efficiencies of health systems could give an incomplete picture of the external challenges and opportunities a healthcare system has to deal with. That is, for an aggregate health output such as life expectancy, a country could perform better than another with the same characteristics, including proper use of factors, just because it has better environmental assets that improve air quality and contribute to reducing deaths. Contrarily, it is more challenging to foster health in an area where air pollution is endemic and a proportion of the population does not have access to adequate sanitation facilities. Yet the relationship between environmental quality and health is well documented in the literature. For instance, Ebenstein et al. [8] claim that China’s modest longevity gains (compared to its neighbours) at the beginning of this century was due to the poor environmental quality, that acted as a countervailing force. They show that a rise of 100 µg/m^3^ in particulate matter concentration in the air results in a 1.5 year shorter newborn lifespan in that country, all else equal. Hence, a health system performance assessment that leaves aside the environmental dimension may underestimate the healthcare system efficiency of countries facing harsh environmental conditions, or otherwise, overestimate the efficiency of countries with a good environment.

In this paper, we analyse the effects of environment quality on the healthcare system performances of OECD countries. Moreover, our aim is to highlight the changes in health system rankings and performances when taking into account the environment, with respect to the WHO-like healthcare system performance league table.^1^

Our starting point will be the seminal analysis provided by the WHO in 2000, which has generated a lot of interest in health system efficiency (see Tandon et al. [5] and WHO [4]). This analysis offered a ranking of 191 national health systems over the period 1993–1997. Although the WHO paper only included health expenditure and education as health inputs, the critics first focused on the estimation method, and rapidly suggested strong enrichment. That is why Hollingsworth and Wildman [6], with the same data, used alternative estimation methods such as data envelopment analysis (DEA) or stochastic frontier analysis (SFA). By doing so, they relaxed the underlying assumption of the panel data model used by the WHO, that is, at least one country is perfectly efficient. However, the criticisms about time-invariant efficiency and the sample heterogeneity were not addressed.

As Hollingsworth and Wildman [6] mentioned, OECD countries, mainly defined as rich countries, could have a different health behaviour compared with other countries. For example, Joumard et al. [9], using simple panel data regression and DEA, argued that healthcare resources do not produce the same “value for money” across OECD countries, but they neglected heterogeneity in behaviour. We depart from these papers by assuming that a part of a country’s inefficiency depends on some random factors, introduced into the SFA model. In that respect, we are close to Ogloblin [7], who analysed health system performances in 78 countries. He included in a SFA model a wide range of health inputs and made the distinction between factors that could directly influence health status (total health spending, the level of education, lifestyle factors), and those that mainly affect the process of producing health, not health status itself (such as income, the share of public health spending in total health expenditure).

In the same vein, de Cos and Moral-Benito [10] investigated the effect of health system institutional arrangements (the capacity of patients to choose an insurer, the scope of basic health insurance, the presence of healthcare price regulation and so on) on their performances for 29 OECD countries. To that purpose, they firstly built a time-invariant SFA model to assess health system efficiencies. Afterwards, they regressed health system characteristics on the obtained efficiency scores. The same type of two-step approach has been used more recently by See and Yen [11] to explore the contribution of people’s happiness towards the healthcare system performances of 121 countries. They carried out a DEA analysis from which they extracted countries’ efficiency scores. The latter was regressed in a second step against a happiness index. Even though the two-step approach is fairly intuitive and popular, especially in the hospital efficiency literature, the validity of this method remains questionable [12–14]. As Kumbhakar et al. [15] suggested, that approach in two steps is biased due to a model mis-specification at the first step. That is why we use a single-step estimation in this paper. Finally, the channel of environmental quality as an explanatory factor of health system efficiency has not been explored in the literature, to the best of our knowledge.

This paper aims to contribute to the health system efficiency literature by highlighting the role of the environment in the process of producing health. For this purpose, we first build a baseline stochastic frontier model (SFA) with no environmental dimension, that estimates the distance between the actual level of health outcome and the potential maximum level the country can reach given its health inputs. In a second approach, we include environmental quality as an input of the health production process. Then we are able to compare model efficiency score estimations with and without the environmental dimension. Environmental quality is measured by the Environmental Performance Index (EPI) of the Yale Center for Environmental Law and Policy, that assesses countries’ performances in environmental goal attainment [16]. Regarding the health system output, we consider life expectancy at birth for its availability and its fairly intuitive interpretation [9]. After this benchmark model, other health output measures will be reviewed, such as life expectancy at 60 years, potential years of life lost, and avoidable mortality.

Our results indicate that the environment is an important factor when assessing health system efficiencies. Indeed, in the environmental model estimation, the magnitude of the effects of health inputs such as income per capita or health expenditure per capita are modified compared with the environment-free model. In regards to efficiency scores, the WHO-like model, that does not include the environment, underestimates (or overestimates) the performances of countries at the bottom (or top) of efficiency distribution, as these countries display a better (or poorer) ranking and efficiency score in the environmental SFA model. The underlying mechanism is that less well performing countries (in terms of health system efficiency) also display poor environmental quality. Hence, their health frontier, which is the potential maximum level of output, tends to be overestimated. Conversely, well-endowed countries, environmentally speaking, such as Sweden or Denmark, do not perform well in the environmental model in comparison with their scores in the WHO-like model because their estimated frontier is lower than their actual frontier, given their better environmental quality.

The remainder of the paper proceeds as follows. Section “The health production function” describes the theoretical rationale of the stochastic frontier analysis (SFA). The section “determinants of health outcomes” presents the data and the variables used in the model. The “Results” section displays the results on the estimated health production function, the “Discussion” discusses these findings and the “Conclusion” concludes.

## The health production function

The health production function refers to the relationship between a health output such as life expectancy at birth, and the inputs that generated it (health expenditures, and so on). Thus, to characterize that function, a first task is to identify which measures of health system outcomes and inputs to consider. That is based on health economics literature and is the focus of the “Determinants of health outcomes” section. More importantly, it is to describe the manner or the functional form by which inputs and outputs are put together. Stochastic frontier analysis (SFA) is one of the ways of doing this while studying health system inefficiencies.

### A stochastic frontier analysis applied to health systems

**Fig. 1:**
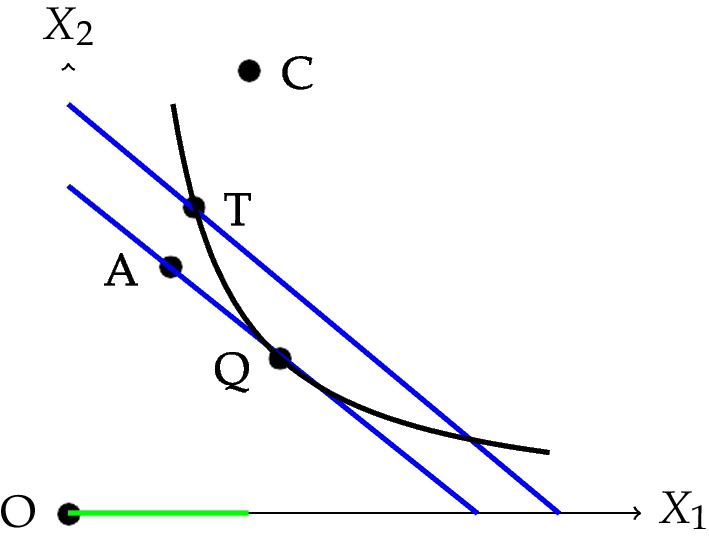
Technical and allocative efficiencies

In this subsection, we present the SFA econometric model, and we define how to measure efficiency. It is worth mentioning that in this paper, we focus on the technical efficiency, that is, the highest level of output health systems can achieve given their inputs, as described in Fig. 1.

Let *y*_*it*_ denote the actual health outcome, for instance life expectancy at birth, and *x*_*it*_ the inputs to the healthcare system. Let us also assume that the health production function can be represented by a Cobb–Douglas function^2^ and that some inefficiencies exist in the process of producing health outcomes. Therefore, the observed health outcome is defined by^3^:

1 $$y_{it} = f\left( {x_{it} ,\beta )\exp (v_{it} - \, u_{it} } \right) = Ax_{it}^{\beta } \exp (v_{it} - u_{it} )$$

*v*_*it*_ represents random errors or shocks that can affect the health production function. They are independent and identically distributed (iid) and follow a normal distribution N (0, *σ*^2^). *u*_*it*_ is the inefficiency term, a non-negative iid term following a truncated normal distribution N ^+^(*µ*_*it*_, *σ*^2^).

Because *u*_*it*_ is non-negative and *v*_*it*_ is a zero-mean error, the (estimated) technical efficiency, *TE*_*it*_, expressed in percentage, is given by^4^,^5^:

$$TE_{it} = exp( - \,\hat{u}_{it} )\quad {\text{where}}\;\hat{u}_{it} \, = \,E(u_{it} |v_{it} \, - \,u_{it} ).$$

The econometric model equation is obtained by a log-transformation of the production function (1):

2 $$Y_{it} = \alpha + \beta X_{it} - u_{it} + v_{it}$$

where *α* = *ln*(*A*), *Y*_*it*_ = *ln*(*y*_*it*_) and *X*_*it*_ = *ln*(*x*_*it*_). Therefore, the estimation for the frontier value is:

$$FE = \exp (\hat{\alpha } + \hat{\beta }X_{it} )$$

The inefficiency in the process of producing health, *u*_*it*_, may depend on a set of other factors *Z*_*it*_ and the mean *µ*_*it*_ can be expressed as *µ*_*it*_ = *λ Z*_*it*_ and we have^6^:

3 $$u_{it} = \lambda Z_{it} + w_{it}$$

where *w*_*it*_ is an iid truncated normal error N (0, *σ*^2^). The truncation point is − *λ Z*_*it*_. *λ *represents inefficiency elasticities with respect to *Z*_*it*_.

### Scope of the analysis

The core of the paper will consist in making two successive estimations of the SFA econometric model described in the section “A stochastic frontier analysis applied to health systems”, with and without the consideration of the environment in the analysis. The model without the environment will be called model 1, while the model with is model 2. We are not intending, with this work, to analyse the causal relationship in the process of producing health at the country level. As is common in the efficiency literature, estimated parameters of the model refer to association and not causation [10, 12, 14]. Another assumption underlying efficiency analyses is that the process of producing the output does not differ a lot across the countries analysed [6, 20]. Since our sample is made up primarily of rich countries at advanced stages of development, we can assume that this assumption is justified [14, 21]. This was one of the main criticisms made of the WHO seminal analysis that included 193 countries, with very heterogeneous healthcare systems [6, 12].

Another important question is what distinguishes variables in the frontier and in the inefficiency term. Ogloblin [7] argues that the former should have a direct impact on the output, whereas factors having an indirect effect on health output are relegated in the inefficiency term. Even though this definition is widely accepted, it is still not clear while reviewing the literature which variables to include in the inefficiency term or in the frontier [10, 14, 22, 23]. In this paper, we will follow the classification by Ogloblin [7], even though this does not rule out a further analysis on variables selection.

## Determinants of health outcomes

In this section, we describe the components of the health production function, as known from previous studies, and afterwards, we give some descriptive statistics of all variables.

### Health inputs in the literature

Factors that directly affect health outcomes are included in the frontier analysis. These variables influence the process of production of health [7]. In the spirit of a SFA, we also have to include variables that enter as inputs of the healthcare system, but affecting health outcomes only indirectly, by acting on system performances. Bearing this distinction in mind, the following variables could potentially influence the level of health system outcomes and are therefore included in the frontier analysis.

*Health expenditures* It is quite evident that the amount of money devoted to healthcare is a direct determinant of health. The larger it is, the higher the life expectancy at birth is in a given country. It is expressed in per capita in current PPP.^7^

*Education* It is well established in the literature that education is a causal factor of the health status [24–26]. In short, a high education level is associated with better outcomes as educated people are more likely to adopt healthier behaviours such as having a healthy diet and exercising. Education is measured in this analysis for each country by the average number of completed years of education of the population aged 25 years and older.

*Alcohol consumption* There is a causal relationship between alcohol consumption and health status. In 2016, the harmful use of alcohol was responsible for 3 million deaths around the world [27]. It is also associated with an increased risk of diabetes, stroke and deaths due to cardiovascular diseases [28]. Hence, the higher the alcohol consumption, the lower the health system outcome. It is measured by the number of litres of pure alcohol consumed by an adult in a year. In addition to alcohol consumption, we could have considered the use of tobacco as a lifestyle determinant of health. However, due to the lack of data, we have not included this variable in our analysis.

*Obesity prevalence* There is also considerable evidence in the literature that obesity is associated with an increased risk of diabetes, high blood pressure and cardiovascular diseases. It affects not only physical but also mental health [29]. Therefore, we can expect lower longevity in a country associated with a higher prevalence of obesity. The prevalence of obesity is defined in this analysis as the percentage of the population with a body mass index (BMI) larger or equal to 30 kg/m^2^.

Some factors are also important for health because they shape healthcare system performances; they must be included in the SFA as possible determinants of efficiency/inefficiencies. They comprise the following variables.

*GDP per capita* There is a consensus in the literature that income is positively related to health status; this becomes trivial when studying the socioeconomic gradient in health at the microeconomic level (comparing households in between). However, at the macroeconomic level, income per se may not affect health directly, but, preferably, by way of giving more efficiency to healthcare spending.^8^This is because having higher incomes gives access to better sanitary conditions, better functioning of the inputs of the healthcare sector and could finance cutting-edge medical facilities, with an influence on healthcare spending efficiency. That is why this variable is included in the inefficiency term, as in [30] or [7].

*Share of public health expenditure* This is the main indicator of the importance public authorities grant to health. However, the effect of the share of public health expenditure in total health spending on health measures is not clear from the literature. For instance, while Linden and Ray [31] find a positive relationship between the share of public health spending and life expectancy, Berger and Messer [32] instead suggest the first is associated with an increased mortality. However, Greene [12] finds no statistically significant effect of the share of publicly financed health services on healthcare production efficiency. The two effects could compete. On the one hand, a greater share of public health spending is associated with more access to health, especially for people at the bottom end of income distribution. On the other hand, it is also related to more administrative burdens, complexity and inefficiency. On this point, we are agnostic and we will rely on data to show the effect on health.

*Out-of-pocket expenditure* Measured by the share of total health spending supported by households, it is also an important feature of a country’s health system. A higher share of out-of-pocket expenditure is associated with lower health system output as it could discourage the demand for healthcare, especially for low-income individuals. Therefore, it could affect the efficiency of healthcare systems.

*EPI* In model 2 (see later), we will also include the Environmental Performance Index, constructed by the Yale Center for Environmental Law and Policy. This will be presented in a special section.

### Data treatment

The data used in this paper come primarily from the OECD statistical database. The share of public health expenditure has been drawn from the World Development Indicators (WDI) of the World Bank. The WHO database has provided the prevalence of obesity for each country. The mean years of schooling for each country comes from the UNESCO database. Our sample is made up of all OECD countries, except Colombia, that joined the organization in 2020. That corresponds to 36 countries observed from 2007 to 2016. Except for variables expressed in percentage or in a 0–100 scale, all the variables included in the model have been log-transformed to interpret parameter estimations as elasticities. Some of them had a few missing values and we used a simple country linear trend model to impute variables. This applies to around 5% of observations in our database.

Estimations were made using STATA V14. The database is available in csv format by clicking this weblink: “dataset” (see with the technical service of Springer Nat)”.

### Descriptive statistics

For a first glimpse at the health production process, we present some summary statistics of all the variables included in the stochastic frontier analysis in Table 1. Over the period of study, life expectancy at birth is on average 80 years, and varies from 70.8 years in Latvia in 2007 to 84.1 years in Japan in 2016. These figures indicate a health output distribution skewed towards lower values, and correspond to developed countries. Thus, on average, the GDP per capita is around US$ 36 000 and can even reach more than three times this amount in Luxembourg.

**Table 1:** Summary statistics

Variables	Obs	Mean	Std. dev	Min	Max
Life expectancy at birth, years	360	79.72	2.84	70.8	84.1
GDP per capita	360	35 910	13 603	14 728	92 302
Health exp per capita	360	3236	1617	733	9904
Share public health exp	360	73.03	9.62	43.64	86.46
Out-of-pocket expenditures	360	20.56	8.77	7.89	52.50
Alcohol consumption	360	9.28	2.70	1.3	14.8
Obesity prevalence	360	21.34	5.70	2.9	36.2
Average years of schooling	360	12.03	1.67	6.27	14.81

It is noteworthy that Mexico, the poorest country over the period of study, does not perform well in terms of health system outcomes (with a life expectancy at birth of just 74.5 years). In addition, it is one of the countries with the lowest health expenditure per capita, maybe because the share of health expenditure supported by patients is very high (52.5%). The United States, the country with the highest health spending per capita (US$ 9904) but a longevity below the average (78.6 years), slightly decreased their high out-of-pocket health expenditure during the period of observation, as the Affordable Care Act came into effect in 2014. Before that reform, the United States was in the lower tail of the distribution of the share of public health expenditures.

Regarding lifestyle, the United States is the country where obesity is the most prevalent (36.2%) whereas the Japanese are the slimmest of our sample (2.9%). Estonia, with 14.8 L of pure alcohol consumed by an adult in a year, is the country with the highest alcohol consumption in our sample. This is associated with a lifespan well below the average, just 76 years. Finally, with 14.81 years of schooling on average in 2015, Canada is the country with the highest level of education, and this is associated with a life expectancy at birth of 81.3 years over the period, above the sample average.

### The environmental performance index

There is a large body of literature, both theoretical and empirical studies, that supports the existence of a positive relationship between environment quality and health [2, 3, 33, 34]. The environmental performance index (EPI) is an indicator that was produced in 2008 by the Yale Center for Environmental Law and Policy that evaluates and ranks countries with respect to their performance in environmental goal attainment. The index is made up of two components: environmental health and ecosystem vitality [16]. The first component measures threats to environmental health and includes indicators such as fine particles exposure and access to improved sanitation or drinking water. The ecosystem vitality gauges natural resources and ecosystem services. It encompasses indicators such as the percentage of forest lost, the intensity of methane and CO_2_ emissions and the percentage of species living in a protected area [16].

The EPI ranges from 0 to 100, with 100 being the highest possible score in achieving environmental goals. Over the period 2007–2016, the average score is 83.43, with a standard deviation of 5.61 (Table 2). The EPI distribution is concentrated and the minimal score, reached by Turkey in 2008, is rare. This country is also the least well performing with respect to the environment in 2015 and 2016. The top five countries of the EPI distribution in 2015 (Finland, Iceland, New Zealand, Sweden, the United Kingdom), all have an above-average life expectancy. Let us also note that the United States is ranked outside the top 20, both in 2015 and in 2016, largely due to their poor performance in terms of ecosystem vitality.

**Table 2:** Summary statistics of the environmental performance index

EPI	Obs	Mean	Std. dev	Min	Max
2007–2016	360	83.43	5.61	59.74	90.86
2015	36	84.27	5.44	66.25	90.74
2016	36	84.51	5.37	67.68	90.68

## Results

### The validity of SFA models

Prior to estimating efficiency models, we have to check whether SFA models are suitable for our data. In other words, we test whether this model brings more meaningful information compared with a standard model, especially the ordinary least squares (OLS) simple linear model. From Equation (2), we can deduce that if the stochastic model is the right one, then the residuals of a standard OLS model (with *X*_*it*_ as independent variables) are skewed to the left, given the asymmetry of the distribution of inefficiency *u*_*it*_.

**Fig. 2:**
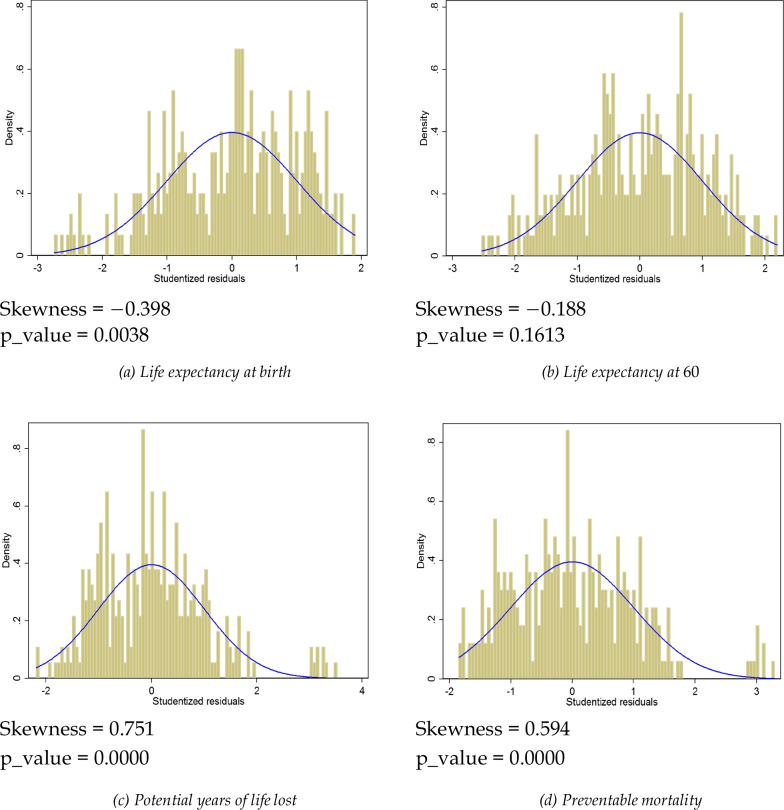
The normal distribution and OLS model residuals

The residuals of the simple OLS model are plotted in Fig. 2a, as well as the density of a standard normal distribution. The skewness of residuals distribution is −0.398, indicating skewness to the left, which is confirmed by the *P*-value (0.0038) of the skewness test. This validity test depends on the health output indicator, life expectancy at birth. We will check other health outcomes at the end of this section, by following the same approach.

### The SFA model estimation – the baseline model

Table 3 reports the results of the stochastic frontier analysis (Equation 2). First, in a model with no environmental dimension (model 1), alcohol consumption, the prevalence of obesity and the amount of resources a country has devoted to health are the main determinants of the frontier of the health production function. A rise by 1% of the number of litres of pure alcohol consumed in a year is associated with a decline by 0.018% of the longevity on average in our countries sample over the period 2007–2016. This represents 5 days of life lost for a country with an average lifespan. Similarly, being overweight shows a detrimental effect on the maximum potential health output, as a 1-percentage-point increase of the prevalence of obesity goes along with a fall by 0.001% of the longevity. Concerning health spending, the more a country invests in health, the more its health output is improved. An increase by 1% of health expenditure is associated with a rise by 0.011% of the frontier of life expectancy at birth. Last, in our regressions made on macrodata, it seems that the level of education is not an important factor for the health outcome, as it is not statistically significant. This may be because our sample is made up of rich countries, that almost all have a high level of education. Therefore, education would not appear as an explanatory factor of the observed differences in life expectancy across countries.

**Table 3:** Estimates of the stochastic frontier model with out-of-pocket expenditures

Dependent variable: life expectancy at birth			
	Model (1)	Model (2)	Model (3)
*Frontier*			
Alcohol consumption	−0.018^∗∗∗^(−6.98)	−0.024^∗∗∗^(−8.99)	−0.023^∗∗∗^(−7.88)
Health exp per capita	0.011^∗∗∗^(3.00)	0.009^∗∗∗^(2.57)	0.013^∗∗∗^(3.39)
Obesity prevalence	−0.001^∗∗∗^(−6.16)	−0.001^∗∗∗^(−8.84)	−0.001^∗∗∗^(−6.66)
Years of schooling	0.107 (0.62)	−0.094 (−0.52)	0.058 (0.33)
Years of schooling^2^	−0.029 (−0.79)	0.013 (0.35)	−0.020 (−0.55)
EPI		0.001^∗∗∗^ (6.45)	
Constant	4.286^∗∗∗^ (19.78)	4.446^∗∗∗^ (19.81)	4.353^∗∗∗^ (19.47)
*Inefficiency*			
GDP per capita	−0.184^∗∗∗^ (−9.51)	−0.171^∗∗∗^ (−8.88)	−0.144^∗∗∗^ (−8.59)
Share public health exp	0.006^∗∗∗^ (5.42)	0.007^∗∗∗^ (5.95)	0.006^∗∗∗^ (5.94)
OOP	0.005^∗∗∗^ (4.50)	0.006^∗∗∗^ (5.20)	0.006^∗∗∗^ (5.34)
EPI			−0.002^∗∗∗^ (−4.17)
Constant	1.338^∗∗∗^ (6.39)	1.103^∗∗∗^ (5.38)	1.047^∗∗∗^ (5.97)
Number of obs	360	360	360
Wald test	108.68	168.90	119.21

*, ** and *** indicate that the coefficient is significant, respectively, at 10%, 5% and 1%

Regarding health system inefficiencies, all factors are statistically significant to explain healthcare system performance across countries. The level of income seems to be the main factor of the performance of health systems. In our sample, the richer a country is, the more efficient its healthcare system is, over the period of study. Wealthy countries benefit from more medical facilities, both public and private, and they are well equipped in terms of cutting-edge medical technology that preserves life. Conversely, a higher share of out-of-pocket expenditure in total health spending is related to lower performances of healthcare systems. Indeed, this could dissuade ill people, especially the poor, to go to hospital when necessary. They consult a health service only when they are at an advanced stage of the disease, with a reduced chance of recovery. Surprisingly, a wider public health system is associated with more inefficiencies. This result may support the criticism of public intervention that creates inefficiencies by overlooking individual preferences, and that suffers from a lack of innovation compared with the private sector.

As aforementioned, these results may depend on the choice of the health output measure. Some critics have said that life expectancy at birth is not a good health measure because it encompasses a lot of the country history and does not give the full picture of recent health system improvements. We will test other health output measures at the end of this section.

As a benchmark, model 1, like Tandon et al. [5], does not include environmental quality, that the literature considers as an important determinant of health. Hence, taking it into account may change the efficiency estimation or the maximum attainable health output.

### Taking environment into account

Starting from model 1, but in view of including the environment in the frontier analysis, we can add the environmental performance index (EPI) either in the frontier (model 2) or in the inefficiency term *u*_*it*_ (model 3). Table 3 indicates that both models are statistically significant to explain the countries’ life expectancy at birth over the period of study. Putting aside the EPI, the same factors highlighted by model 1 remain significant when the environmental quality is considered, even though the magnitude of their effects is modified.

#### Environmental models

The EPI is statistically significant in both models 2 and 3. This means that environmental quality is a relevant factor when studying OECD countries health system performances. With EPI in the frontier, a ten-point increase of the environmental quality index goes along with a rise by 0.01% of life expectancy at birth. This represents 3 days of longevity gain for a country like France.^9^ Therefore, all else equal, a country that preserves ecosystems and promotes renewable energy can reach a higher maximum health output. On the other hand, with the environmental quality in the inefficiency term, the same policy reduces the health system inefficiency.

Turning to health inputs effects, the elasticity of health output with respect to alcohol consumption is greater in models 2 (0.024) and 3 (0.023), compared with model 1. Obesity prevalence has the same effect on health whether the environmental quality is included or not, whereas compared with the model without environmental quality, the effect of health spending is lower in model 2 (0.009) and higher in model 3 (0.013). The effects of education are still negative, decreasing and not statistically significant even when the environment is considered.

Regarding the inefficiency term, including environment lessens the elasticity related to GDP per capita. From 0.184 in the model with no environmental consideration, it falls to 0.144 in model 3. The magnitude of the effects of the share of public expenditure and of out-of-pocket expenditure are almost unchanged from model 1 to models with environmental quality. Let us also mention that including the environmental quality mainly affects the income effect in the inefficiency term, and alcohol consumption and health spending in the frontier. Finally, the frontier productivity factor is greater in environmental models compared with the model with no environment dimension. We have the opposite pattern with the inefficiency-related productivity factor.

#### Environment in the frontier or in the inefficiency

Now we have to choose which model best takes into account environmental quality. In other words, is assuming that environmental quality affects the frontier more consistent with data, compared with assuming that the environment influences the health systems inefficiencies? To answer this question, we have to check the amount of information brought by each model. This is measured by information criteria, where the most used are the Akaike and Bayesian information criteria (AIC and BIC, respectively). The more meaningful model is the one that has the lowest value for the criterion considered. Therefore, Table 4 suggests that it is the model with environment quality affecting the maximum potential output that better fits our data, either using AIC or BIC. Hence, it is model 2 that we will use in the next subsection to assess healthcare system performances when environmental quality is included.

**Table 4:** Model comparison statistics with life expectancy at birth

Dependent variable: *life expectancy at birth*			
	log-likelihood	AIC	BIC
Model 1	897.24	−1770.48	−1725.11
Model 2	917.22	−1808.44	−1759.29
Model 3	904.14	−1782.28	−1733.13

### Ranking healthcare system efficiencies

**Fig. 3:**
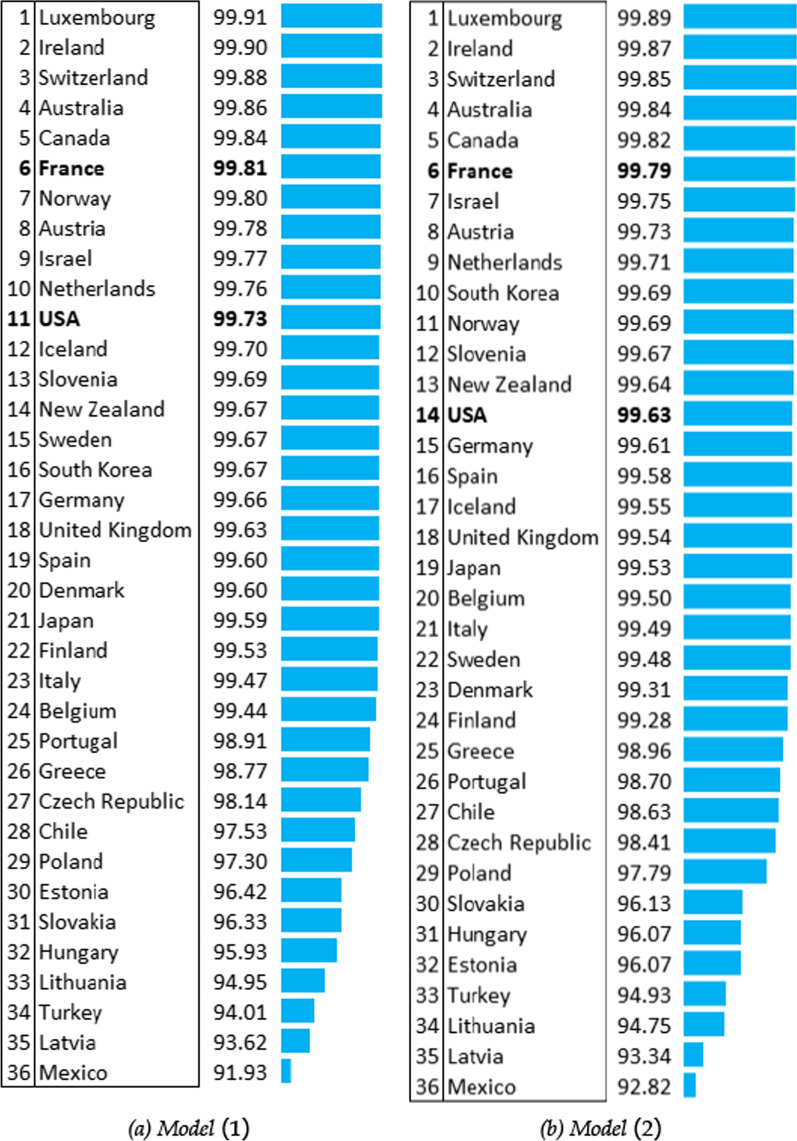
Health system efficiencies (%) with life expectancy at birth as health output in 2016

Figure 3 ranks OECD countries with respect to their health system efficiency scores in 2016, with and without environmental quality considered. We have chosen the 2016 estimations because it is the latest available year. For each of the two econometric models, we have the corresponding ranking along with histograms in descending order of the heath system efficiency. The figure suggests that countries at the top are close in terms of health performance whether the environmental dimension is added or not, although sizable changes start to occur at the middle and at the bottom of the distribution. From the figure, we also learn that health system efficiencies decrease more rapidly at the bottom of the distribution.

*The environmental model – model 2* The league table derived from the environmental model displays a more concentrated distribution of health system performances compared with the one from the model without environmental consideration: efficiency scores range from 92.82% to 99.89%. Comparing the rankings of model 2 with model 1, 11 out of 36 countries have the same ranking and 15 countries climb in the league table when environmental quality is included. In the same time, only ten countries have a higher health system efficiency score in the environmental model. In short, it seems that adding the environment mainly modifies the inefficiency gap in the middle of the distribution, and that countries at the bottom tend to gain slightly in terms of (measured) efficiency. For instance, the United States falls by three positions in the ranking and France has the same ranking with a lower efficiency score. In the same time, Belgium, previously 24th, gains four places. The greatest rank improvement comes from South Korea (+6), whereas Scandinavian countries are the most disadvantaged by the environmental model, with Sweden (−7), Norway (−4) and Denmark (−3). Mexico, Turkey and Poland are at the bottom in model 1 but tend to gain efficiency in model 2 (while still remaining at the bottom of the ranking).

Until now, we have considered life expectancy at birth as the measure of the health system output. The main advantages of this indicator are its availability and its quite intuitive interpretation. However, there is a debate in the literature on the best indicator that captures the global health status of a population, and each potential candidate presents some flaws [9]. That is why we consider other health output indicators for SFA models in the next subsection.

### Other health outcomes

One could have chosen other available health indicators to use in the SFA, such as life expectancy at 60, potential years of life lost, and avoidable mortality. In this section, we rapidly comment on the results when using such alternatives.

*Life expectancy at 60 years* With this health output, the residuals of a standard linear OLS model are skewed towards the left, as indicated by the skewness statistics equal to −0.188 < 0 (Fig. 2b). Contrary to the case with life expectancy at birth, all the variables in the SFA model are statistically significant factors of the health output (Table 5), including education (with a quadratic term). In the inefficiency term, the effects of income and of the share of public expenditure are greater compared to the SFA model with longevity at birth.

The environmental model with the EPI in the frontier becomes the preferred model, because best supported by the data (Table 8). With this health output, health systems are overall less efficient in 2016 than previously, as just around 10 countries display less than 1% of inefficiency compared with around 25 in the rankings with life expectancy at birth (Fig. 5). Similarly, the least performing country in the environmental-free model has a much lower efficiency score, 82.95% compared with 91.93%. It is also noteworthy that including the environmental dimension results in a more concentrated distribution of efficiencies. It would seem that the environment-free model overestimates the health performances of countries at the top of the ranking, and underestimates those of countries at the bottom. Sweden, Finland and Denmark are the most penalized by adding the environmental dimension, whereas Mexico are Turkey are the most advantaged.

*Potential years of life lost (PYLL)* This indicator attributes a higher weight to younger age deaths. Unlike the previous health output indicators, the PYLL is a negative output of the health system, that is, the smaller it is, the more efficient the healthcare system. Hence, a SFA model with this indicator would be valid if the residuals of the standard linear OLS model are skewed towards the right, which is the case here with a skewness statistic of 0.751 > 0 (Fig. 2c).

As with longevity at birth, the SFA model still suggests a negative relationship between the average years of schooling and the health status, even though education is not statistically significant (Table 6). All the other health inputs in the frontier are relevant to explain the difference in PYLL across countries over time. Including the environment in the frontier is the best environmental model as suggested by Table 9. Turning to the health systems ranking in 2016, the best performing country is Luxembourg, in which the observed PYLL is 1.79% greater than the minimal possible level of output according to the environment-free model (Fig. 6). Countries at the bottom and at the top of the league are almost the same than as with the other health output indicators. However, it is noteworthy that the United States is now among the bottom five countries in terms of health system efficiency, whatever the model considered. Belgium (+9) and South Korea (+7) are the countries whose rankings improve the most when moving from model 1 to the environmental model. At the other end of the scale, like in the previous subsection, Sweden (−7) and Denmark (−8) rankings fall the most.

*Avoidable mortality* According to the OECD, it is the number of deaths that could be avoided through public health and prevention intervention. Like PYLL, the smaller it is, the better it is for the national healthcare system. The residuals of the linear model are skewed towards the right, with a skewness statistic of 0.594 > 0 (Fig. 2d).

In the environment-free SFA model, the prevalence of obesity does not appear significant (as well as the frontier intercept), unlike the model with other health output measures (Table 7). The level of education is significant and the return to education is negative and increases with the years of education. Last, including the environment mainly lessens the importance of income in the health production process. Incidentally, with preventable mortality as a health output, the environmental model that is best supported by the data is model 3, that considered that environmental quality directly affects health system inefficiencies (Fig. 10).

Concerning the health system performance league table in 2016, Luxembourg still has the most efficient health system (Fig. 7). Comparing the environmental model with the environment-free benchmark model, the bottom five remain the same, whereas Switzerland and Italy are pushed out of the top five in 2016 in the environmental model. In the same way, except for Turkey and Mexico, all the countries in the bottom ten of the (in)efficiency scale come closer to their frontiers when environmental quality is taken into account. In contrast, only four countries in the top ten reduce the gap with their minimal potential health output in the environmental model.

**Fig. 4:**
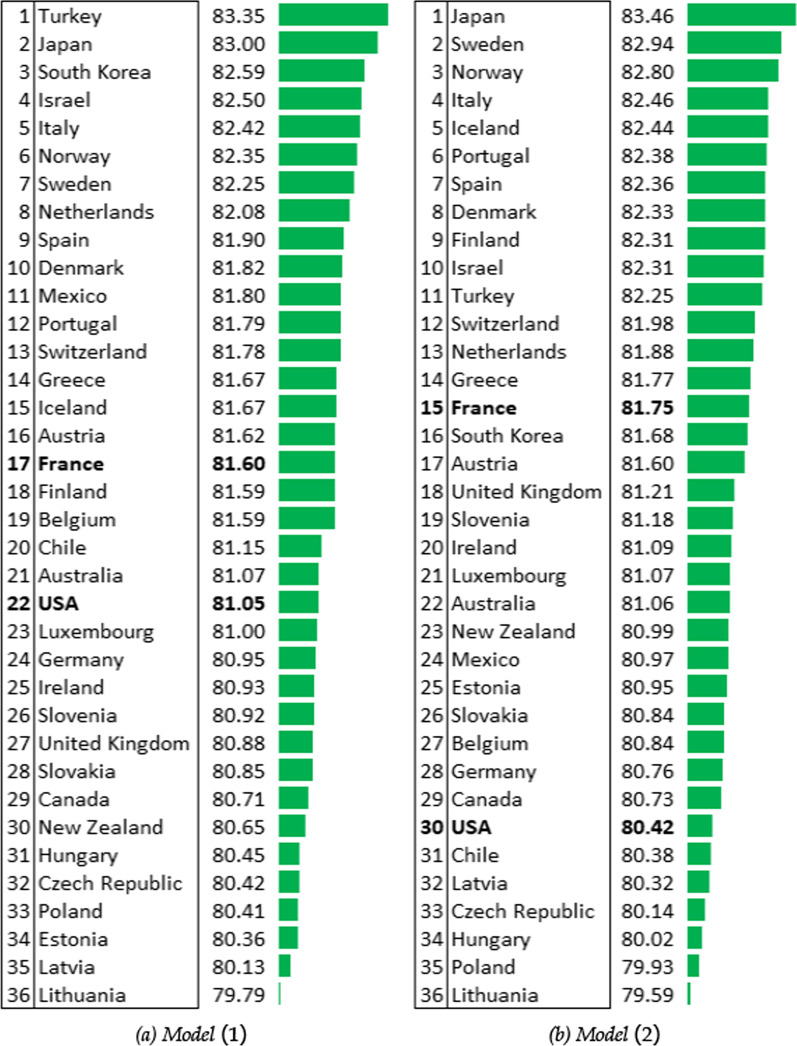
The frontier of the health system (in years) with life expectancy at birth as the health system output in 2014

Once again, it seems that not taking into account the environment underestimates the healthcare system performance of countries at the bottom of the efficiency distribution, since those countries are those doing poorly in terms of environmental quality. Indeed, the correlation coefficient between health system ranks in the environment-free model and the variation in the environmental model is positive, as presented in Table 11, whatever the health system output indicator. In a consistent way, the maximum potential health output of countries such as Mexico and Turkey, is overestimated in the baseline model because it does not take into account the harsher environmental conditions these countries have to cope with (Figs. 4, 8, 9, and 10).

Until now, we have just described and presented the results of the stochastic frontier analysis. In the “Discussion” section, we will analyse and discuss these results to highlight the message from including environment in analysing health systems performances.

## Discussion

In this paper, we introduced a multicountry–multiperiod indicator of environment quality in a stochastic frontier analysis of the OECD health systems, and we tested how it impacts their measured performances. We obtained that the indicator was highly significant as a direct factor of the populations’ health outcome and that the rankings of countries in terms of health system efficiency could be substantially modified by these considerations. The foundation of the paper can be linked to the “One Health” approach, defined by the WHO as “an approach to designing and implementing , policies, legislation and research in which multiple sectors communicate and work together to achieve better public health outcomes” [35]. The One Health approach particularly emphasizes the multiple interactions that human health has with the environment for the cases of respiratory diseases due to zoonoses (of which COVID 19 is probably one [36]), but in general it recognizes that the health of people is closely interconnected to the health of the planet [37], or at least to the quality of the local environment where people live [38]. An interconnection we confirm in the framework we use, and this could be the main finding of our own contribution.

As expected, better environmental quality is associated with higher health system outcomes, whether the EPI is included in the frontier or in the inefficiency term. When the EPI is considered as a health frontier determinant, a country with a higher environmental quality displays a higher maximum potential health output compared with the environment-free model, all else equal. By contrast, a country with harsh environmental conditions has a lower health frontier in the environmental model. Among variables included in the model, the GDP per capita is the one whose coefficient changes the most (by decreasing) when the environmental dimension is taken into account. Through the data analysis, we probably have a substitution effect in the main channels by which population health is created: the effects on health of some environmental quality components such as the level of pollution, heavy metal soil contamination or forest preservation, that were correlated with country incomes, now go through the environmental index.

We can also go a little further in the study of health system performances: let us first note that all the nonenvironmental variables included in the inefficiency term are significant to explain differences in country performances. Considering life expectancy at birth as the health output, larger shares of healthcare directly paid by households and the relative importance of the public health sector are associated with more inefficiencies. Indeed, greater out-of-pocket expenditure may discourage people’s demand for healthcare. The negative association between the size of the public health sector and health system efficiency may be explained by the failures of a government-run system to properly take into account individual preferences or by the complexity and the administrative burden associated with the public sector. However, the relationship becomes positive with PYLL or preventable mortality as health system outputs, maybe because a well-developed public health sector goes in tandem with better access to health for disadvantaged people, who are also more difficult to care for. Equity will then compete with (’measured’) efficiency.

Regarding the health system performance league table, Western European countries such as Luxembourg, France and Norway are well represented in the top of the ranking. In contrast, Eastern European countries such as Hungary and Latvia performed poorly in terms of health system efficiency in 2016, both in the baseline and the environmental models. That could be explained by the significant weight of GDP per capita as estimated by both models. However, as already noted, the effect of income is mitigated once the environment has been included in the picture. That is why some top-performing countries in the baseline model such as Luxembourg and Switzerland have lower efficiency scores in the environmental model. Let us also note that countries with high environmental quality such as France and Australia are well represented at the top of the scale of the efficiency score in the baseline model. On the contrary, Turkey, Mexico, Hungary and Poland, that are not doing well in terms of environmental quality, are also those improving their ranking the most in the environmental model, compared with the baseline one. We find the same relationship when it comes to the distance to the frontier, that is, countries with highest efficiency scores are those which move furthest away from their frontier in the environmental model compared to the baseline one. Therefore, a ranking from an environment-free model, like the one used by the WHO, overestimates performances of countries endowed with good environmental quality, and belittles those with poor environmental quality, because these unlucky countries do not benefit from the environmental conditions that favor high health output levels.

This research has some limitations that should be acknowledged. The main limitation is probably the duration of the analysis period (10 years). A longer duration would not only have offered more statistical power in the analysis, but also allowed the introduction and study of more lagged effects, with the EPI in particular. Discussions could be opened about the selection of variables that are used in the SFA model: both inputs and outputs, although we decided to follow the conventional literature and the available data. For the inputs, the mix of money-assessed variables and of quantitative ones is debatable, but it was impossible to have only quantitative inputs when doing such a large scale international comparison (for example, counting the number of GPs, specialists, nurses, etc. is a nonsense since they do not have the same roles and missions depending on the healthcare system). To discuss the issue of the appropriate outcomes, we simply decided to alternate various candidates, studying whether there were large changes associated with certain outcomes (as a sensitivity analysis).

## Conclusions

The aim of this analysis was to study the effect of the environment on health system efficiency for OECD countries over the period 2007–2016. For that purpose, we estimate and compare stochastic frontier models with and without an environmental dimension. We find that the environment, when taken into account, is a major determinant of the health production function as it significantly affects the elasticities related to other health factors such as obesity, alcohol or the GDP per capita. Country health system ranking and efficiency scores are also altered. Compared with an environmental model, a baseline model with no environmental dimension underestimates countries at the bottom of the distribution. That is because such a model does not take into account the harsher environmental conditions least performing countries have to cope with to produce health. This analysis once again underlines the critical importance of the environment, for both policy-makers and researchers, when tackling health issues.

**Fig. 5:**
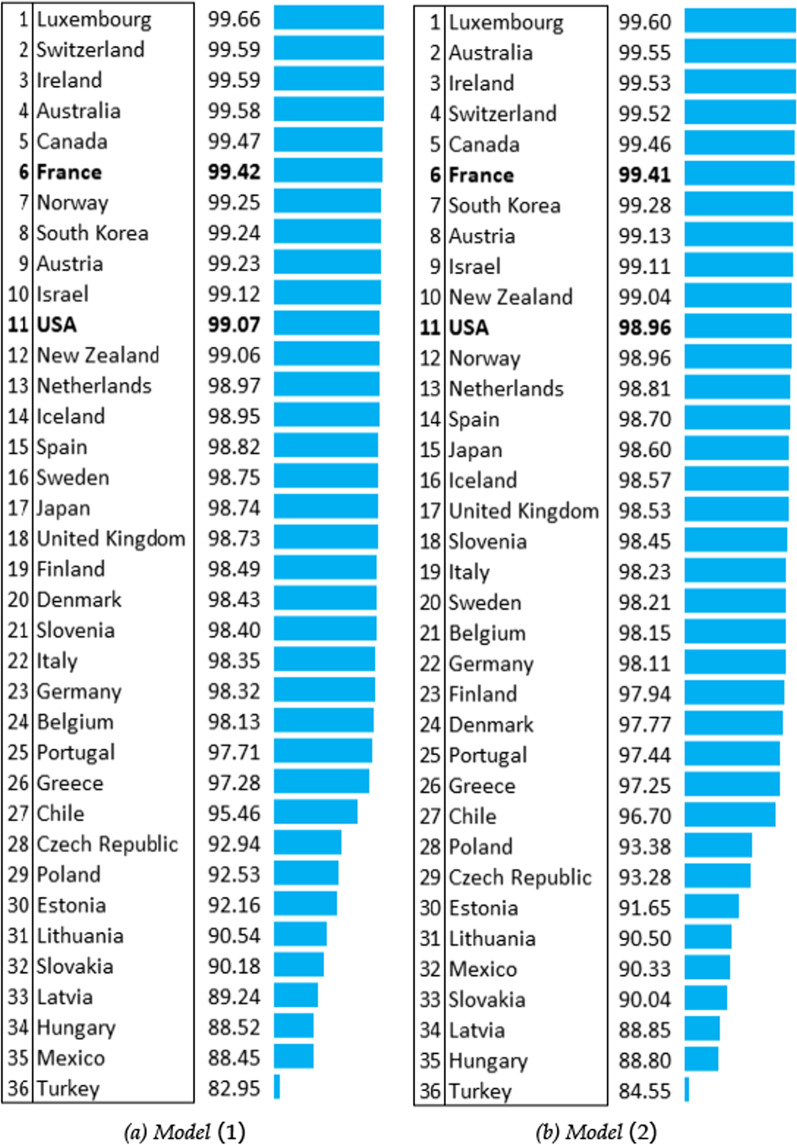
Health system efficiencies (%) in 2016 with life expectancy at 60 years as health system output

**Fig. 6:**
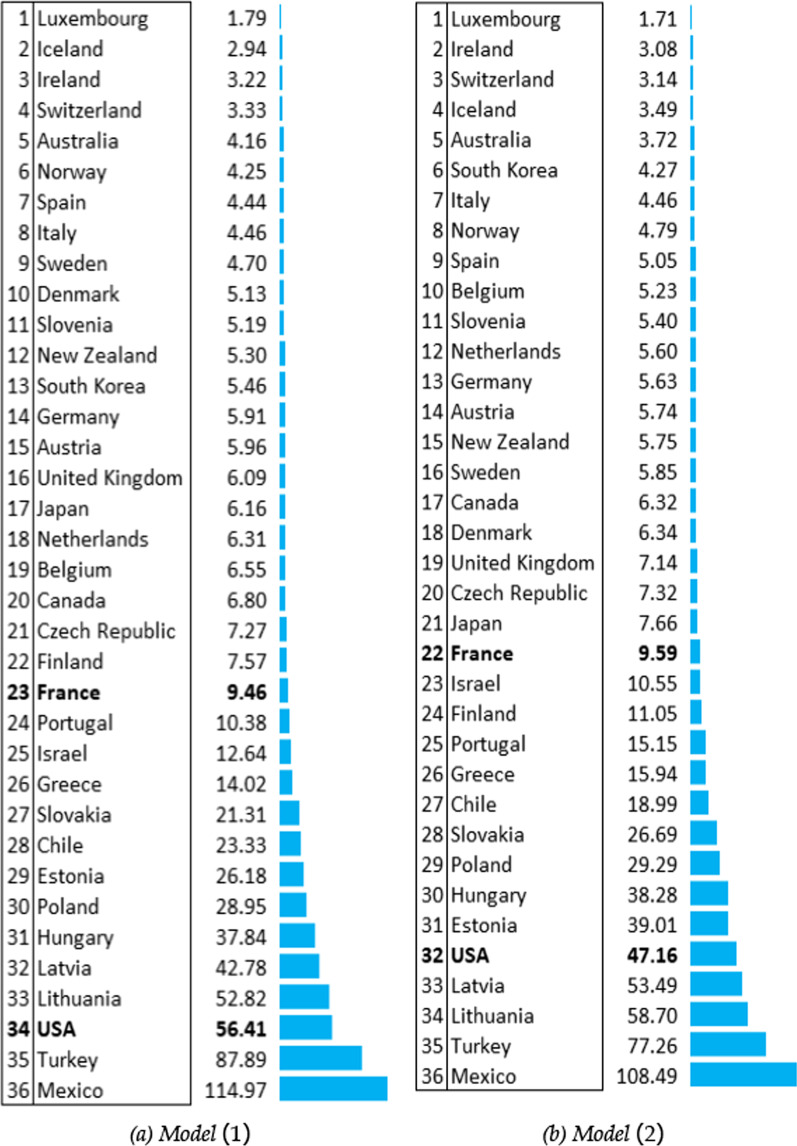
Health systems inefficiencies (%) in 2016 with potential years of life lost as health system output

**Fig. 7:**
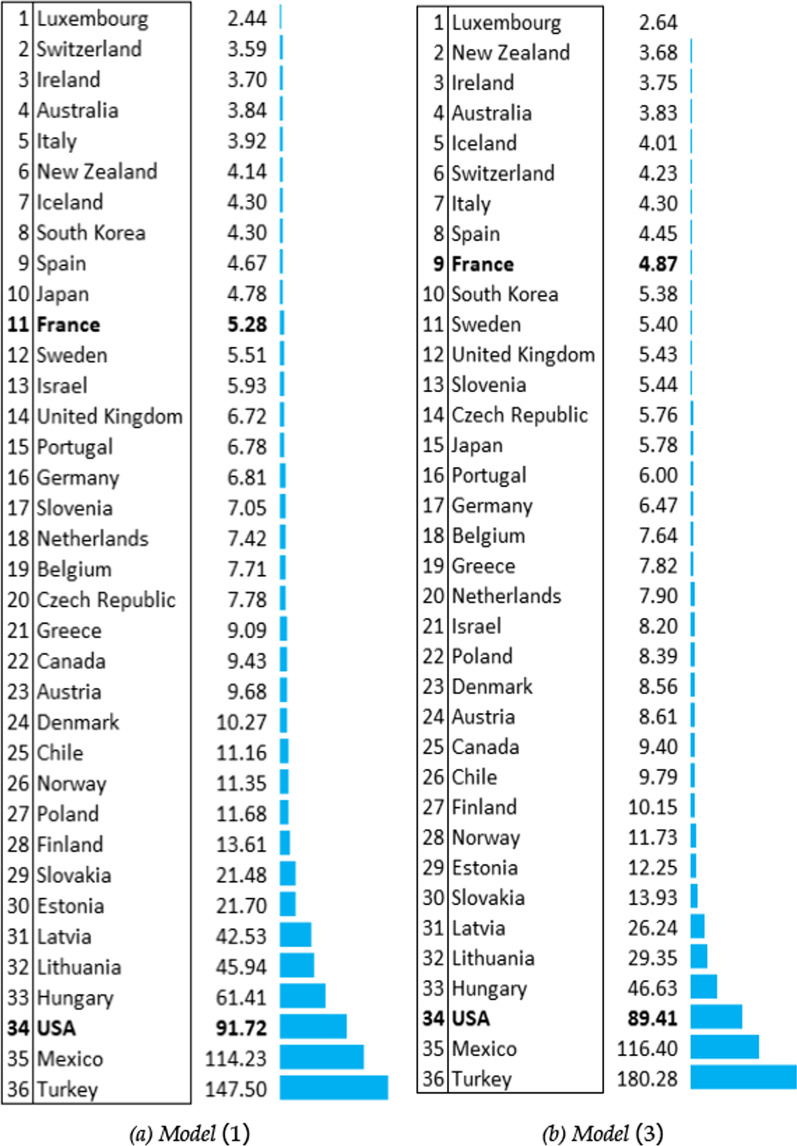
Health system inefficiencies (%) in 2016 with preventable mortality as health system output

**Fig. 8:**
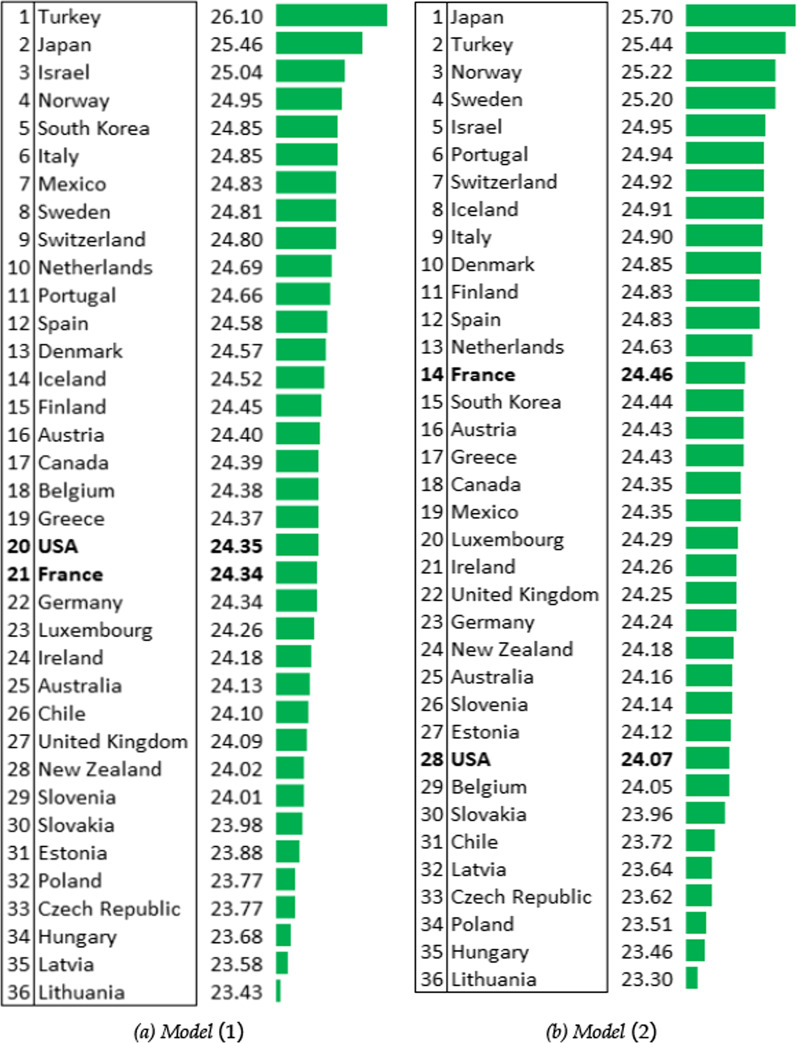
The frontier of the health system (in years) with life expectancy at 60 years as the health system output in 2016

**Fig. 9:**
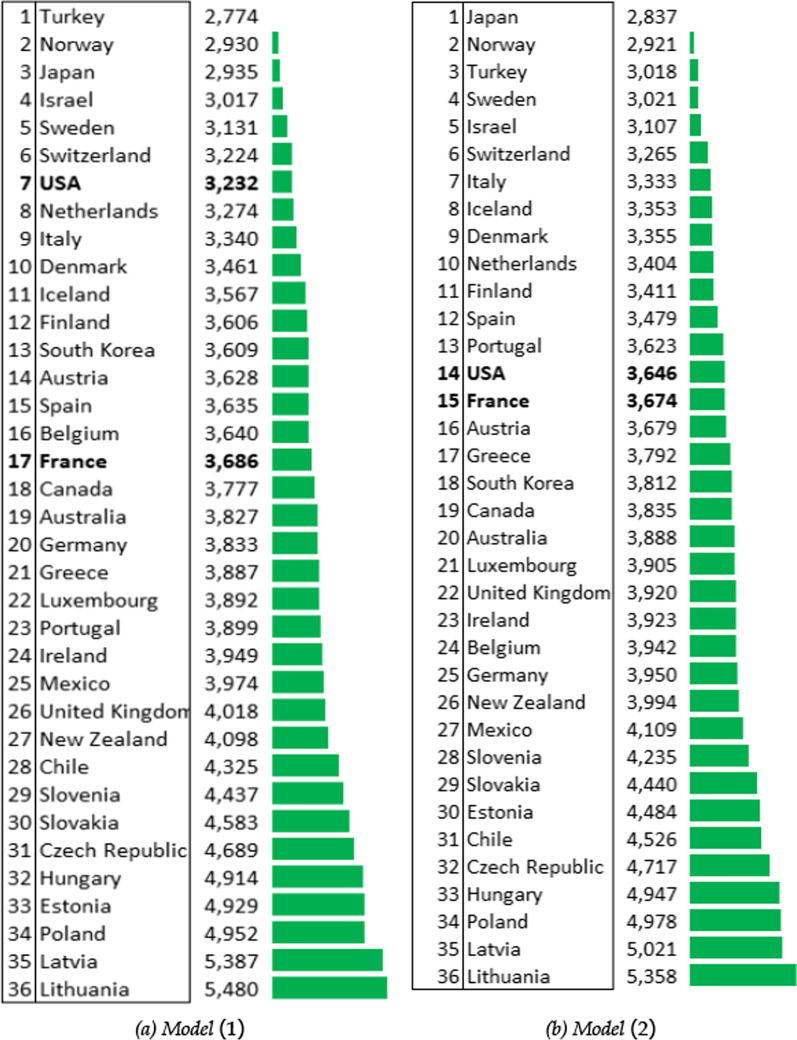
The frontier of the health system with potential years of life lost as the health system output in 2016

**Fig. 10:**
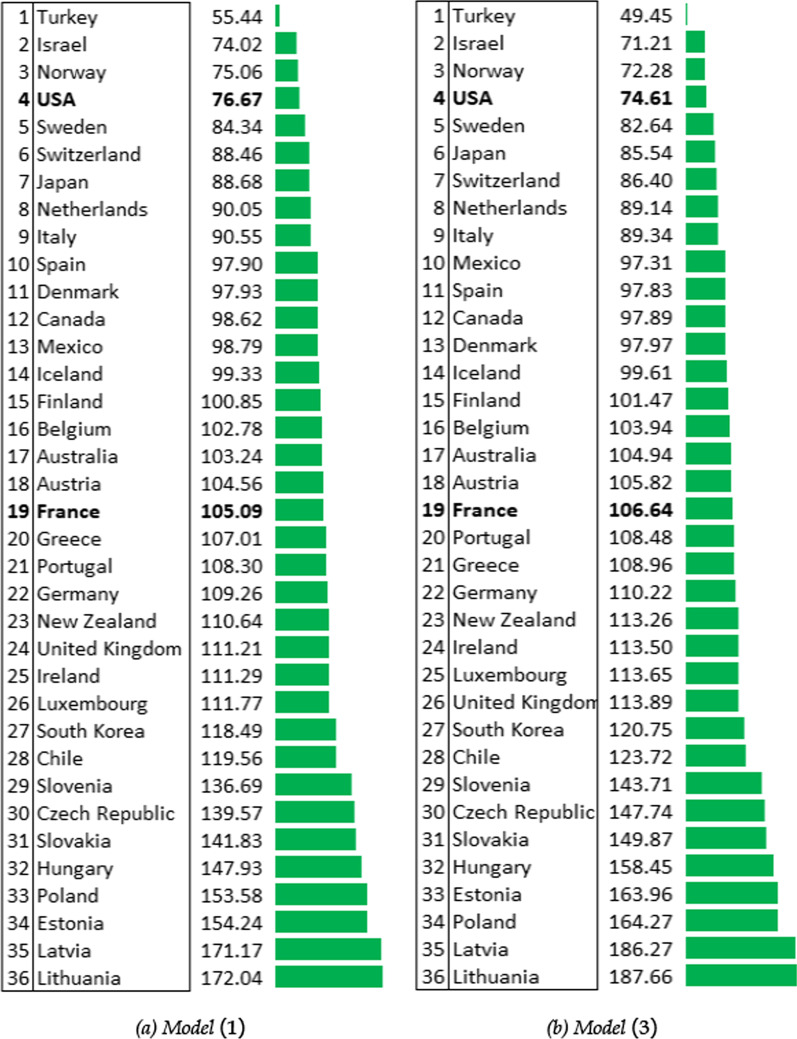
The frontier of the health system with preventable mortality as the health system output in 2016

See Tables 5, 6, 7, 8, 9, 10, 11 and Figs. 5, 6, 7, 8, 9, 10

## Data Availability

Data are available on internet (free of charge)

## Appendix

In this figure  ,^10^ the actual input mix (*X*1, *X*2) is at *C*, while the output level is equal to the one at *T*. The health system is inefficient because it can produce the same level of output with the input mix at *T*. Therefore, the technical efficiency can be measured by the ratio: *TE* = *OT*/*OC*.

The allocative efficiency implies producing the same level of output at the lowest cost. It can be measured by: *AE* = *OA*/*OT*.

See Tables 5, 6, 7, 8, 9, 10, 11 and Figs. 5, 6, 7, 8, 9, 10

**Table 5:** Estimates of the stochastic frontier model with life expectancy at 60 years

Dependent variable: life expectancy at 60			
	Model (1)	Model (2)	Model (3)
*Frontier*			
Alcohol consumption	−0.037^∗∗∗^ (−5.43)	−0.047^∗∗∗^ (−6.49)	−0.046^∗∗∗^ (−5.97)
Health exp per capita	0.024^∗∗^ (2.53)	0.022^∗∗^ (2.41)	0.030^∗∗∗^(2.93)
Obesity prevalence	−0.002^∗∗∗^ (−4.28)	−0.002^∗∗∗^ (−5.67)	−0.002^∗∗∗^ (−4.59)
Years of schooling	−1.028^∗∗^ (−2.38)	−1.174^∗∗∗^ (−2.67)	−1.166^∗∗∗^ (−2.66)
Years of schooling^2^	0.201^∗∗^ (2.23)	0.231^∗∗^ (2.52)	0.226^∗∗^ (2.47)
EPI		0.002^∗∗∗^ (3.96)	
Constant	4.430^∗∗∗^ (8.19)	4.463^∗∗∗^ (8.06)	4.597^∗∗∗^ (8.36)
*Inefficiency*			
GDP per capita	−0.426^∗∗∗^ (−8.79)	−0.378^∗∗∗^ (−7.55)	−0.349^∗∗∗^ (−7.28)
Share public health exp	0.012^∗∗∗^ (4.74)	0.014^∗∗∗^ (5.03)	0.012^∗∗∗^ (4.60)
OOP	0.007^∗∗∗^ (2.72)	0.009^∗∗∗^ (3.27)	0.007^∗∗∗^ (2.93)
EPI			−0.003^∗∗∗^ (−2.89)
Constant	3.354^∗∗∗^ (6.50)	2.725^∗∗∗^ (5.10)	2.845^∗∗∗^ (5.96)
Number of obs	360	360	360
Wald test	83.28	105.57	81.50

*, ** and ***Indicate that the coefficient is significant respectively at 10%, 5% and 1%

**Table 6:** Estimates of the stochastic frontier model with potential years of life lost

Dependent variable: potential years of life lost			
	Model (1)	Model (2)	Model (3)
*Frontier*			
Alcohol consumption	0.321^∗∗∗^ (11.35)	0.338^∗∗∗^ (11.69)	0.371^∗∗∗^ (10.37)
Health exp per capita	−0.293^∗∗∗^ (−5.93)	−0.223^∗∗∗^ (−4.87)	−0.312^∗∗∗^ (−4.80)
Obesity prevalence	0.008^∗∗∗^ (5.39)	0.012^∗∗∗^ (7.15)	0.009^∗∗∗^ (5.44)
Years of schooling	0.653 (0.38)	2.439 (1.35)	1.831 (1.05)
Years of schooling^2^	−0.068 (−0.19)	−0.440 (−1.17)	−0.303 (−0.83)
EPI		−0.011^∗∗∗^ (−4.66)	
Constant	8.527^∗∗∗^ (3.92)	6.593^∗∗∗^ (2.90)	7.084^∗∗∗^ (3.23)
*Inefficiency*			
GDP per capita	−1.224^∗∗∗^ (−5.45)	−1.059^∗∗∗^ (−5.74)	−0.994^∗∗∗^ (−5.19)
Share public health exp	−0.031^∗∗∗^ (−4.31)	−0.021^∗∗∗^(−3.54)	−0.029^∗∗∗^ (−3.66)
OOP	−0.029^∗∗∗^ (−3.67)	−0.019^∗∗∗^ (−3.02)	−0.026^∗∗∗^ (−3.34)
EPI			−0.016^∗∗∗^ (−2.52)
Constant	15.446^∗∗∗^ (5.59)	12.877^∗∗∗^ (5.84)	14.157^∗∗∗^ (5.82)
Number of obs	360	360	360
Wald test	197.51	241.57	145.51

*, ** and *** Indicate that the coefficient is significant respectively at 10%, 5% and 1%

**Table 7:** Estimates of the stochastic frontier model with preventable mortality

Dependent variable: preventable mortality			
	Model (1)	Model (2)	Model (3)
*Frontier*			
Alcohol consumption	0.457^∗∗∗^ (15.70)	0.456^∗∗∗^ (16.40)	0.525^∗∗∗^ (16.56)
Health exp per capita	−0.392^∗∗∗^ (−10.31)	−0.309^∗∗∗^ (−6.52)	−0.450^∗∗∗^ (−14.48)
Obesity prevalence	0.002(1.47)	0.005^∗∗∗^ (3.16)	0.003^∗^ (1.90)
Years of schooling	3.737^∗∗^ (1.96)	2.943(1.57)	5.756^∗∗∗^ (3.09)
Years of schooling^2^	−0.660^∗^ (−1.68)	−0.518 (−1.34)	−1.058^∗∗∗^ (−2.75)
EPI		−0.009^∗∗∗^ (−3.60)	
Constant	1.608 (0.69)	2.720 (1.15)	−0.638 (−0.28)
*Inefficiency*			
GDP per capita	−2.751^∗∗∗^ (−2.76)	−1.448^∗∗∗^ (−3.19)	−1.441^∗∗∗^ (−3.42)
Share public health exp	−0.071^∗∗^ (−2.51)	−0.030^∗∗^ (−2.20)	−0.059^∗∗∗^ (−3.86)
OOP	−0.078^∗∗^ (−2.40)	−0.031^∗∗^ (−2.08)	−0.057^∗∗∗^ (−3.69)
EPI			−0.057^∗∗∗^ (−3.48)
Constant	34.392^∗∗∗^ (2.77)	17.538^∗∗∗^ (3.13)	24.275^∗∗∗^ (4.30)
Number of obs	360	360	360
Wald test	269.05	313.99	384.843

**, ** and ****Indicate that the coefficient is significant respectively at 10%, 5% and 1%

**Table 8:** Model comparison statistics with life expectancy at 60 years

Dependent variable: life expectancy at 60 years			
	log-likelihood	AIC	BIC
Model 1	594.88	−1165.75	−1120.39
Model 2	602.83	−1179.65	−1130.50
Model 3	598.52	−1171.03	−1121.88

**Table 9:** Model comparison statistics with potential years of life lost

Dependent variable: potential years of life lost			
	log-likelihood	AIC	BIC
Model 1	154.79	−285.58	−240.21
Model 2	165.79	−305.58	−256.43
Model 3	159.04	−292.08	−242.93

**Table 10:** Model comparison statistics with preventable mortality

Dependent variable: preventable mortality			
	log-likelihood	AIC	BIC
Model 1	131.28	−238.56	−193.19
Model 2	136.99	−247.98	−198.84
Model 3	143.19	−260.39	−211.24

**Table 11:** Spearman correlation coefficients

	Rank	Efficiency score
Life expectancy at birth	0.10	−0.09
Life expectancy at 60 years	0.00	−0.14
PYLL	0.17	0.01
Preventable mortality	0.08	0.48

The column “Rank” gives the Spearman correlation coefficient between countries’ ranks according to the baseline model, and the number of places gained or lost in the environmental model. For instance, the ranking of the United States health system is 11th and the variation in ranking in the environmental model is −3, as they are at the 14th position according to that model.

The column “Efficiency score” gives the same correlation coefficient between efficiency scores in the baseline model and the variation in the distance to the frontier in the environmental model

## Footnotes

1 See WHO [4].

2 For the sake of simplicity, a Cobb–Douglas production function has been chosen. One could have assumed a more general function such as a transcendental logarithmic one and the underlying rationale still holds.

3 See Battese and Coelli [17].

4 $\hat{\mu}$ means an estimated value.

5 *u*ˆ_*it*_ = *E*(*u*_*it*_* v*_*it*_*u*_*it*_) is the estimator of Jondrow et al. [18].

6 See Battese and Coelli [19].

7 PPP: Purchasing power parity.

8 We tested the alternative view of introducing gross domestic product (GDP) per capita in the frontier (as a direct factor); this alternative manner was statistically dominated, giving a *P*-value > 0.05 for the GDP per capita variable.

9 The estimated health frontier for France is 81.7 years.
